# Effect of Cold Atmospheric Plasma on Epigenetic Changes, DNA Damage, and Possibilities for Its Use in Synergistic Cancer Therapy

**DOI:** 10.3390/ijms222212252

**Published:** 2021-11-12

**Authors:** Dušan Braný, Dana Dvorská, Ján Strnádel, Tatiana Matáková, Erika Halašová, Henrieta Škovierová

**Affiliations:** 1 Biomedical Centre Martin, Jessenius Faculty of Medicine in Martin, Comenius University in Bratislava, 036 01 Martin, Slovakia; dusan.brany@uniba.sk (D.B.); jan.strnadel@uniba.sk (J.S.); erika.halasova@uniba.sk (E.H.); henrieta.skovierova@uniba.sk (H.Š.); 2 Department of Medical Biochemistry, Jessenius Faculty of Medicine in Martin, Comenius University, Bratislava, 036 01 Martin, Slovakia; tatiana.matakova@uniba.sk

**Keywords:** cold atmospheric plasma, epigenetic changes, non-coding RNA, synergistic treatment, DNA damage

## Abstract

Cold atmospheric plasma has great potential for use in modern medicine. It has been used in the clinical treatment of skin diseases and chronic wounds, and in laboratory settings it has shown effects on selective decrease in tumour-cell viability, reduced tumour mass in animal models and stem-cell proliferation. Many researchers are currently focusing on its application to internal structures and the use of plasma-activated liquids in tolerated and effective human treatment. There has also been analysis of plasma’s beneficial synergy with standard pharmaceuticals to enhance their effect. Cold atmospheric plasma triggers various responses in tumour cells, and this can result in epigenetic changes in both DNA methylation levels and histone modification. The expression and activity of non-coding RNAs with their many important cell regulatory functions can also be altered by cold atmospheric plasma action. Finally, there is ongoing debate whether plasma-produced radicals can directly affect DNA damage in the nucleus or only initiate apoptosis or other forms of cell death. This article therefore summarises accepted knowledge of cold atmospheric plasma’s influence on epigenetic changes, the expression and activity of non-coding RNAs, and DNA damage and its effect in synergistic treatment with routinely used pharmaceuticals.

## 1. Introduction

Plasma is the fourth fundamental state of matter. It is assumed that 99% of the observed mass in the universe is composed of some type of plasma [1]. Although plasma is most often defined as an ionised gas, this definition is inaccurate in several respects. Plasma is transient in nature and depends strongly on the behaviour of charged particles, and especially on electrons. The ionised gas must have the following three criteria to satisfy the plasma definition [2,3,4]. (1) Quasi-neutrality. The plasma must contain almost the same density of positively and negatively charged particles. Therefore, in the absence of external disturbances in plasma, the net resulting electric charge is roughly zero. (2) Debye shielding. When an external point charge is introduced, or unbalanced charge is formed in the plasma, charge density is readjusted to neutralise the effect by shielding its electric field. The mechanism of shielding a foreign charge or an unbalanced charge inside the plasma is known as Debye shielding. The characteristic distance over which electrostatic potentials are attenuated by a redistribution of the charged particles is Debye length. (3) Plasma frequency. The most mobile plasma elements, the electrons, move towards or away from the unbalanced charge to restore the original charge neutrality. This provides oscillation, and the frequency at which these oscillations take place is known as plasma frequency [2,3,4]. Simplified plasma definition therefore is “a quasi-neutral gas containing many interacting free electrons and ionised atoms and molecules which have collective behaviour caused by long-range coulomb forces” [4].

Plasma is most often categorised by the motion of the particles and their interaction within its volume, and these defines its final temperature. This provides three plasma categories—high temperature, thermal and non-thermal [4,5]. (1) High-temperature plasma has electrons and heavy particles in thermal equilibrium. This enables plasma to achieve its highest temperature up to 10^8^ °K. For example, stars are almost entirely ionised balls of high-temperature plasma; (2) Thermal plasma does not have particles in thermal equilibrium in its entire volume, only smaller volumes in thermal equilibrium. This plasma can be up to 2 × 10^4^ °K and is found, for example, in lightning; and (3) non-thermal plasma does not have particles in thermal equilibrium. Non-thermal plasma is usually found in artificially created light devices, and although it can have temperatures up to 1000 °K, it is called cold or non-thermal plasma because its temperature can be as low as 300 °K [4,5].

Despite the low temperature and low level of ionisation, cold plasma generates various active species, such as reactive oxygen (ROS) and nitrogen (RNS) species and charged particles, UV radiation, and localised electric fields. The reactive species can be formed by plasma interaction with either liquid or air [6,7,8,9]. Due to these properties, it has become a subject of interest in several areas, including medicine. Cold plasma can be generated under low pressure and also under atmospheric pressure [10]. Low pressure generation has limited use in medicine, primarily in instrument sterilisation, and surface modification of implants and tissue engineering products [11]. In contrast, cold atmospheric plasma (CAP) has a wider range of use. It can be used for decontaminating surfaces from prokaryotes, viruses and prions [12,13,14]. Moreover, it has already been used successfully in clinical practice for skin regeneration and other dermatological treatment in addition to its important function in chronic wound healing [6,15,16]. In experimental conditions, it has strong anti-proliferative effect on tumour cells under in vitro conditions and tumour tissues in animal models in vivo, with only mild impact on healthy cells [14,17,18,19,20,21,22]. It is also useful in promoting stem-cell proliferation [23,24,25,26].

However, although CAP is generally considered a safe option for use on healthy eukaryotic cells, minor and temporary drawbacks have been reported. An example of this is the focal mucosal erosions in mouse models accompanied by ulceration and necrosis lasting a few days after CAP exposure [27]. Moreover, higher doses of ROS and RNS produced by CAP can potentially have negative effects on morphological and haemolytic changes in red blood cells [28]. This strongly indicates that CAP must always be applied in correct dosage and duration, and also with appropriate voltage, pulse shape, frequency and distance [29,30,31,32].

CAP generating devices are divided into two types, depending on whether the generated plasma discharge is applied directly or indirectly to target structures [33,34,35]. Direct discharge is usually generated as a dielectric barrier discharge (DBD) with the affected structures placed between the grounded and high voltage electrodes. However, there are several variations of DBD devices. For example, either one or both electrodes can be covered with a dielectric layer, or a dielectric barrier can be placed between the electrodes. The most suitable DBD sub-type for clinical practice is the floating electrode DBD (FE-DBD) which uses the affected structure as a grounded electrode.

Indirect discharge is generated by devices which first generate a plasma discharge between two electrodes, and a carrier gas then directs discharge flow. The devices are called plasma pens, jets or torches, and the carrier gases are usually noble gases such as helium or argon. In addition, there are also experimentally tested hybrid plasma devices which combine the benefits of both direct and indirect application, but none are yet approved for commercial use [33,34,35].

Although liquids or media can also be exposed to CAP and subsequently applied to cells or tissues, these are only experimental and not yet used in routine treatment. The plasma-activated media (PAM) and liquids (PAL) such as Ringer’s lactate solution or plasma-activated water (PAW) have different properties to those that are un-activated, mainly due to their higher ROS and RNS content. Moreover, long-term species including nitrates, nitrites and hydrogen peroxide (H_2_O_2_) can be preserved in PAM and PAL [36,37,38]. PAM, PAL and PAW have been reported to affect the cells and tissues they contact, and they can also decrease tumour cell lines’ viability and reduce tumour mass in animal models under in vivo conditions [38,39,40,41,42,43]. Therefore, several possibilities of applying CAP exist, and these are expected to increase in the future. For example, there is current research to improve methods of plasma application to subdermal and internal structures. This is based on designing a miniature but sufficiently powerful CAP device which can act similar to a needle or an endoscope to penetrate the dermis or enter human cavity-areas and apply plasma into subdermal and internal structures [44,45,46,47,48]. A further option would be to improve PAL and PAW application so that it is safer, more tolerable and effective for future human use [41,49].

This especially applies to PAW, and its most achievable use could be an antimicrobial mouthwash to eliminate typical oral pathogens such as *Streptococcus mutans*, *Actinomycetes viscosus*, *Porphyromonas gingivalis*, and *Enterocococcus faecalis*. This is possible because PAW eliminates biofilms and bacteria are not resistant to PAW [41,50,51]. Moreover, research has also proven its safety, without negative effects on animal model tissues [52]. PAW has also strong antiproliferative effect on various cancer cell lines [43]. Oral PAW administration may therefore be helpful in mouth and upper digestive tract tumour treatment [41].

Although CAP has been the subject of interest in medicine for less than 20 years, William Crookes had already introduced it for the first-time in artificial conditions in the 19th century. It was then almost 50 years before Irwin Langmuir named it and described its properties in greater detail in 1927 [53]. Most studies in the past 20 years have focused on describing CAP’s anti-cancer effects which are mostly based on plasma’s generation of ROS and RNS. In addition, researchers also focused on CAP’s effect on the generation of nitric oxide (NO), because this is crucial for the induction of stem-cell proliferation [14,17,18,19,20,21,22,23,24,25,26].

Although normal cells can relatively successfully defend themselves against harmful ROS and RNS effects by activating anti-oxidative systems [54], cancer cells have much higher ROS levels [55], and altered mechanisms which regulate redox balance [56]. Further CAP elevation of ROS and RNS levels can be therefore fatal for tumour cells, while healthy cells suffer minimum damage [56]. There is also a theory that tumour cells are more susceptible to CAP treatment because they replicate more often. Tumour cells therefore enter the S phase of the cell cycle more frequently. In S phase is DNA un-winded and more susceptible to ROS and RNS [57,58]. However, although cancer cells are more sensitive to the oxidative stress from reactive species and radicals generated by CAP [59], the response varies depending on stressor concentration. For example, H_2_O_2_ can either stimulate cell proliferation or induce apoptosis [60]. Although NO inhibits protein oxidation and lipid peroxidation, and also diminishes membrane permeability and limits apoptosis at low concentration, it causes cell injury at higher concentrations [61].

Lipid peroxidation is a typical result of CAP-cell interaction, and it comprises a cascade process, where oxidants and free radicals attack lipids in cell. This especially involves lipids containing carbon double-bonds, such as those present in polyunsaturated fatty acids [62,63,64]. Healthy cells under low lipid peroxidation rates are normally able to stimulate antioxidant defence mechanisms by up-regulating antioxidant proteins. This provides appropriate adaptive stress response. However, high lipid peroxidation can overwhelm cell repair mechanisms, and induce apoptosis or necrosis [62,63,64]. There are the following three recognised lipid peroxidation steps [63]; (1) pro-oxidants, such as the ROS and RNS produced by CAP, abstract the allylic hydrogen forming the carbon-centred lipid radical (L•); (2) the second step is propagation where L• reacts with oxygen to form a lipid peroxy-radical (LOO•) which abstracts a hydrogen from a further lipid molecule to generate the primary lipid hydro-peroxide product (LOOH), and a new L• which continues the chain reaction; and (3) the third step involves antioxidants which donate a hydrogen atom to the LOO• species to form a corresponding radical which reacts with another LOO• to produce non-radical products. However, secondary peroxidation products can form if the cell is unable to terminate this process. The secondary products are mainly aldehyde degradation entities, and their cell toxicity leads to cell signalling defects or cell death when they are in high concentration. Typical aldehyde degradation products are 4-hydroxy-2-nonenal (4-HNE) and malondialdehyde (MDA) [62,63,64]. 4-HNE is considered a secondary messenger of oxidative stress, and its high concentrations induce death in most tumour cells [65]. However, some hepatoma cells were observed to successfully degrade those peroxidation products, and those cells suffered no further damage [65].

Cell membrane lipid order can decrease as a result of lipid peroxidation. High peroxidation levels can create membrane pores, and these facilitate increased reactive species’ penetration into cells [62,63,64]. Tumour cells have different membrane lipid composition and cholesterol levels, and this can make them more susceptible to peroxidation [19,66,67,68,69,70,71,72]. The reactive species and radicals which penetrate cell membrane pores can then affect signalling pathways and this can result in signalling defects or cell death [73,74]. However, membrane lipid composition changes vary in different tumour cell types, and some tumour cells are then more susceptible to peroxidation than other tumour or healthy cells [19,65,66,67,68,69,70,71,72].

CAP products can also cause phospholipid bilayer etching [75,76,77,78], and this significantly affects cell membrane structure and function. [76]. This etching is induced mainly by OH radical molecules which oxidise the phospholipid bilayer head-groups. OH radicals interaction with phospholipids resulted in cleavage and dissociation of bonds, and the OH radicals preferentially affected the C-N and C-O bonds [76]. The phospholipid tail-groups were oxidised subsequently. Phospholipid bilayer etching can lead to initial rise of membrane rigidity, followed by strong increase in membrane fluidity [76].

In addition to reactive species and radicals, CAP-generated UV radiation also influences plasma-cell interaction. UV radiation can damage DNA, especially through the formation of pyrimidine dimers, and these increase both cell mutagenicity and cytotoxicity. There are two types of pyrimidine dimers [79]; (1) Cyclobutane pyrimidine dimers are produced when adjacent pyrimidine residues are covalently linked and; (2) pyrimidine (6-4) pyrimidone photo-products are generated when single covalent bond between the carbon at the 6 position of one ring and carbon at the 4 position of the ring on the next base occurs. An accumulation of cyclobutane pyrimidine dimers and 6-4 photo-products can generate mutations and lead to cell death [79]. The CAP generation of variable wavelength UV radiation connected with pyrimidine dimer formation [80] can therefore pose a potential CAP application risk and this highlights that plasma must be applied only in relevant dosage and under regulated conditions. CAP-generated UV radiation and the risk of DNA damage are further discussed in a later section of this paper.

There is still a lack of knowledge how CAP influences epigenetic changes and alters the activity and expression of non-coding RNAs. In addition, there is still divided opinion on the direct effect of CAP on DNA damage. This article therefore summarises the accepted knowledge of how CAP effects these changes, and the possibility of using CAP in synergistic treatment with standard and experimental anti-cancer agents.

## 2. Epigenetic Changes

Although epigenetic changes do not directly affect DNA sequence, they influence resultant gene expression [81]. The primary role of epigenetic changes is specialising the various cell’s body function and ensuring cell-type maintenance [82]. These epigenetic changes are divided into those affecting the methylation levels of nucleic acids and those influencing histone proteins. Finally, the epigenetic status of cancer cells is often impaired [83,84,85,86,87].

### 2.1. DNA Methylation

A methyl group is added to cytosine during DNA methylation. This specifically occurs at the 5th carbon in the pyrimidine ring, and this results in 5-methylcytosine formation [88]. DNA methylation is performed by three DNA methyl transferases—DNMT1, DNMT3a, and DNMT3b. Although DNMT1 maintains methylation status, DNMT3a and DNMT3b are responsible for de novo methylation [88,89,90]. DNMT3a/b obtains a methyl group from the S-adenosyl methionine molecule during the DNA methylation process. Multi-step de-methylation features “ten-eleven translocation methylcytosine dioxygenases” (TET).

The cytosine in the CpG dinucleotides is methylated in mammalian cells, and the regions with a large number of CpG di-nucleotide repeats are referred to as “CpG islands”. These islands can be up to 3,000 bp and are usually located near the gene promoters [88,89,90].

The physiological balance between DNMT and TET enzyme activity is significantly impaired in cancer cells, and this most often results in hypermethylated tumour-suppressor genes [91]. Methylated CpG dinucleotides in promoter regions of the tumour-suppressor genes negatively affect the binding of transcription factors and this results in lower expression of the affected gene.

Although there are several types of epigenetic histone modification [89], this paper describes only CAP effects on histones methylation, acetylation, and phosphorylation. These epigenetic histone changes are mainly chemical modifications of their N-terminal tails which extend from the nucleosome and are therefore more easily accessible [89,92]. In addition, the C-terminal tails [92] or the globular domains [93] can also be modified, but these occur less frequently. The N-terminal tails are recognised by other chromatin proteins and they can form docking sites to facilitate the recruitment of specific non-histone proteins to chromatin [92]. This interaction can then alter chromatin packaging and the final expression of particular genes.

### 2.2. Histone Methylation

Histone methylation can enhance or repress expression [94]. The methylation, however, does not alter charge, and this contrasts sharply with histone acetylation and phosphorylation which changes histone charge [94]. There can be methylated histone lysine and arginine residues. The best characterised are histone 3 lysine 4 methylation (H3K4me), histone 3 lysine 9 (H3K9me) and histone 3 lysine 27 methylation (H3K27me). H3K4me enhances transcription and is usually close to gene promoters, while H3K9me and H3K27me inactivate transcription and are mainly associated with heterochromatin [94,95,96,97,98,99]. However, final methylation effects differ depending on the number of methyl groups bound to the histone residue. Although up to three methyl groups can bind to the lysine residues, the arginine residues can only be mono-methylated, or symmetrically or asymmetrically di-methylated [94,97,98,99].

All the above-mentioned methylations are associated with different methyla-transferases and de-methylases, and Hyun described precisely how these coordinate histone methylation [100]. Histone methylation generates motifs recognised and bound by other proteins, especially those with the Chromo, Tudor, MBT and PHD domains [94,95,96,97,98,99]. This protein binding can subsequently affect final transcription activation or inhibition. Finally, It has also been described that ROS have the ability to change the histone methylation status [101,102].

### 2.3. Histone Acetylation

Histone acetylation involves the addition of an acetyl group to the histone lysine residues. This process is regulated by histone acetylases (HATs) and determines whether chromatin is open or condensed. Acetylation reduces histone positive charge [103,104,105], and it decreases histone attraction to the negatively charged DNA. Histone deacetylation is regulated by deacetylases (HDAC) which enzymatically remove the acetyl group from histone lysine residues, and this facilitates histone binding to the negatively charged DNA. This then leads to chromatin condensation and repression of DNA transcription [106]. Therefore, histone acetylation is generally considered a factor which increases gene expression because it weakens the binding between the histones and DNA, thus making the DNA more accessible. Acetylated groups can also act as a docking site for various proteins, especially those containing bromo-domains [107,108] and this further affects histone packaging and final gene expression. [107,108]. These proteins can re-model chromatin or recruit other proteins which affect chromatin remodelling. Finally, histone acetylation occurs more often than other epigenetic modifications and it is a very dynamic process [105,108].

### 2.4. Histone Phosphorylation

Histone phosphorylation is a modification which can occur on serine, threonine and tyrosine residues, and it is associated with a wide range of cell changes [109]. The phosphorylation is mediated by protein kinases which add a phosphate group, and phosphatases which remove it [110]. Although histone phosphorylation can initiate transcription, it is also associated with chromosome segregation, DNA damage and apoptotic induction [109,110]. There are four canonical histones with several variants [111]. Here, the H2AX histone variant differs from the H2 canonical histone in its carboxyl terminus [111,112]. This variant is highly conserved and can be phosphorylated on serine residue 139. H2AX phosphorylation is primarily associated with DNA damage and double-strand breaks but also includes other cell processes [113,114,115,116]. It is mediated by ATM [117], ATR [118], and/or DNA-PKcs [119] and occurs on nucleosomes on both sides of DSBs at a DNA mega-base domain [120]. The Ser139-phosphorylated H2AX is known as γH2AX. The ‘γ’ signifies that this modification was first noted following gamma irradiation. However, cell physiologic processes such as DNA recombination can be associated with higher levels of γH2AX [121]. Moreover, cells undergoing apoptosis also induce extensive H2AX phosphorylation [122,123,124], and importantly, the amount of γH2AX in apoptotic cells has been found to be 10 times higher than in non-apoptotic cells [125]. In addition, The H2AX phosphorylation is also very dynamic, and approximately 1% of all H2AX proteins are phosphorylated by each irradiation gram [126].

## 3. Non-Coding RNAs

Non-coding RNAs (ncRNAs) form 98% of the total cell transcript, and these have important functions, especially at the post-transcription level. The two types of ncRNAs are housekeeping and regulatory RNAs [127]. Housekeeping RNAs are constitutively expressed in all cell types under physiological conditions, and these include transfer RNAs, ribosomal RNAs, small nuclear RNAs, small nucleolar RNAs, and telomerase RNA. In contrast, regulatory RNAs vary in their expression in different cell types, and their activity is largely dependent on environmental factors [128]. These include long non-coding RNAs (lncRNA) over 200 bp and the short non-coding RNAs which are divided into microRNAs (miRNA), short interfering RNAs and Piwi-interacting RNAs [127,128,129]. This paper describes the CAP effects on miRNA and lncRNA expression.

### 3.1. Micro RNAs

The miRNAs are the most well-known and analysed non-coding RNAs. They have from 21 to 25 bp, and most often 22 bp. Although their genes are mainly transcribed by polymerase II from both intergenic and intragenic regions, some sequences coding miRNA which spread along *Alu* repeats are transcribed by polymerase III [130]. miRNAs from the same family are often transcribed in clusters [131]. These single-family miRNAs have similar seed sequence at the 5′end which is essential for mRNA binding [132].

There have been approximately 1,900 pre-miRNAs and over 2,600 mature mRNAs described in the human genome [133,134]. The miRNA biogenesis pathways can be canonical or non-canonical, and both these pathways and the subsequent inclusion of mature miRNAs into an miRISC complex are described in detail in O’Brien [130] and Jiang et Yan [135]. The main miRNA function is to regulate gene expression on post-transcriptional levels, and this is achieved by the following two mechanisms—the miRISC complex with miRNA can directly cleave mRNA, or that complex can modulate loading of ribosomes [130,135]. The miRNAs most often interact with the mRNA 3′UTR region, but also with the 5′UTR region or directly with the gene promoter region [136], and although their main function is to repress target gene expression, some also enhance gene expression [130,133]. This is usually seen in ‘starved’ cells in non-physiological conditions [137,138]. Finally, miRNAs can have either a tumour-suppressive or oncogenic effect [139].

### 3.2. Long Non-Coding RNAs

The lncRNA sequence is over 200 bp [140], and lncRNAs undergo splicing and removal of intron-like sequences, similar to the genes which encode proteins. Although there are more than 270,000 lncRNAs in the human genome, many of these have very low copy number [141]. Moreover, the lncRNA sequences can be located close to the genes, in the space between genes or even overlapping genes, and their binding varies. This binding can be in the 3′-5′direction, the 5′-3′direction, bi-directional and also to intron and intergenic regions and enhancer sequences [142].

The lncRNAs also have various cell functions and effects. Examples include; they can form a complex with the HNRPNK nuclear matrix protein which is involved in the regulation of structural organization of the chromatin [143,144]; Xist lncRNA is involved in inactivating one of the X chromosome [145] and C0T-1 lncRNA affects chromosome decondensation [146]. Furthermore, they can recruit the PRC2 polycomb repressive complex, and this can lead to the deposition of histone H3 lysine 27 trimethylation (H3K27me3) [147]. Myosin Heavy Chain Associated RNA Transcript lncRNA clusters are antagonistic to the Brg1 catalytic subunit of the BAF chromatin remodelling complex [148]. The lncRNAs can interact with the SWI/SNF complex [149] and they can modify histones by altering their methylation [150] and acetylation [151], or they can affect DNA methylation levels [152,153]. Moreover, lncRNAs can have the function of competing endogenous RNAs in cells [154]. The lncRNAs with this ability contain the microRNA response elements which are complementary to miRNA binding sites, and this results in miRNA binding to this lncRNA instead of binding to the target mRNA sequence.

## 4. CAP Effect on DNA Methylation

Park et al. noted [155] the effect of CAP on the MCF-7 and MDA-MB-231 breast cancer (BC) cell lines. These authors analysed the changes in the methylation levels of the selected CpGs in *Alu* elements which are typically hypermethylated in normal tissues but often hypomethylated in cancer [156]. Their initial pyrosequencing analyses revealed slight hypo-methylation in the selected *Alu* sequence following exposure to CAP, but this occurred only in the MDA-MB-231 cells. Although the 23.4% to 20.3% decrease in methylation rate was statistically significant at *p* < 0.05, it is questionable whether this can be considered important because no statistically significant changes were noted in the second cell line, and 3% is not such a great difference. However, whole-genome methylation microarray analysis in the same cell lines provided more important results. In particular, the 318 CpGs in the MCF-7 cell line were hypermethylated and 56 CpGs were hypomethylated in the CAP-treated cells compared to untreated ones. There were also 76 CpG sites hypermethylated and 63 CpGs hypomethylated in the MDA-MB-231 cells, compared to untreated MDA-MB-231 cells. The list of affected sequences also included the promoter CpG sites of non-coding RNAs, and 17 miRNAs and 25 lncRNAs had significant methylation change. Subsequent qPCR analyses showed that the expression of 9 of the 11 selected genes were associated with the methylation level. These results indicate that CAP involvement most likely has significant effect on DNA methylation changes, especially in cancerous cells. CAP exposure also caused significant tumour-cell structural change. In addition, while the oestrogen-negative MDA-MB-231 tumour cell line had increased apoptosis rate compared to the oestrogen-positive MCF-7 cell lines, there was no apoptotic increase observed in the MCF-10 and MCF-12 non-cancerous cell lines following CAP treatment.

Hou et al. [157] focused on differences in the final CAP effect on A549 small lung-cancer cells related to the length of CAP exposure. The authors treated the cells with CAP for 1 and 3 min and monitored the changes in gene expression 2, 4, and 6 h after this application. The authors noted that cells treated with CAP for 1-min had affected the activity of different groups of genes compared to the cells after 3-min exposure.

The study revealed the following; (1) genes down-regulated after 1-min exposure were mainly related to cellular metabolism, chromosome processes, cell-cycle function and ATPase and nucleoside-triphosphatase activity; (2) genes up-regulated after 1-min exposure were related to kinase and transcription factor activity and ROS response; (3) genes regulating methylation and acetylation remained unchanged after 1-min exposure, and although many important pathways including IL-4, MAPK, TGF, TNF, and p53 mediated signalling pathways were activated by this exposure, this significantly decreased after 3-min exposure; (4) it is important that the altered expression of a significant number of genes that occurred 2 h after 3-min exposure was connected with the changes in methyltransferases activity. Therefore, 3-min-or-longer CAP exposure can potentially affect DNA methylation by altering methyltransferase activity; and (5) 3-min CAP exposure also caused up-regulation of genes important in histone acetylation. Therefore, exposure time can determine the CAP final effect on cell signalling cascades leading to changes in epigenetic status. Finally, it has also been noted that exposure to CAP significantly affects the DNA methylation levels of important genes which regulate the morphology, germination and growth in plants such as rice [158] and soybean [159].

## 5. CAP Effect on Histone Methylation and Acetylation

Lee et al.’s pilot study on CAP’s influence on histone methylation levels used the H3K4me3 genome-wide ChIP-sequencing method [160]. H3K4me3 is associated with activating the expression of nearby genes [94], and the authors showed that up to 899 sequences which fell within the promoter regions had changes in H3K4me3 methylation level in MCF-7 cells after CAP exposure. These sequences were then examined for functional inter-relatedness and the pathways with the highest confidence involved “Cellular Compromise, DNA Replication, Recombination, Repair, and Cell Cycle”. It is also important that the expression levels of 18 selected genes had statistically significant correlation with the extent of altered H3K4me3 methylation level. The authors then verified the *USMG5*, *HSCB*, *PRPS1*, *C15orf48*, and *LRRC28* genes’ expression levels with qPCR. *PRPS1* is considered a BC oncogene [161], and *LRRC28* a potential hepatocellular oncogene [162]. They found that these genes’ expression and H3K4me3 methylation levels were higher in unaffected cells than in CAP exposed cells. The authors’ final demonstration then indicated that CAP action should induce JARID1A demethyltransferase by ROS-mediated cellular signalling. However, elucidation of this interactive mechanism remains necessary.

CAP’s effect on histone acetylation changes has also been demonstrated [163]. Whole-genome RNA sequencing identified statistically significant increase in 469 genes’ expression and a decrease in 941 gene expression following the CAP exposure of adipose tissue-derived stem cells (ASC). Higher activity was especially noted in genes involved in cytokine and chemokine activity, and decreased activity was mostly observed in genes involved in apoptotic pathways. Most importantly, CAP-treated cells also had increased histone deacetylase 1 (HDAC1) activity and decreased acetylated histone-3 levels. The altered HDAC1 expression was most notable 9 h after CAP exposure, but this decreased after 24 h, and expression was then almost the same as in unexposed cells. In contrast, histone 3 acetylation decrease was greatest 24 h after exposure. In addition, cells exposed to CAP, and then subjected to HDAC1 and DNA and histone methylation inhibitors, resulted in lower cytokine and growth factor activity. This enables the assumption that CAP application affects ASC’s via epigenetic changes. Although these results were demonstrated on stem cells, it cannot be excluded that similar mechanisms occur in cancer cells. Further investigations should therefore prove valuable.

The application of HDAC inhibitors to A549 BC cell lines and their cultivation in PAM enhanced PAM-induced cell injury [164]. PAM treatment alone increased the amount of TUNEL positive cells with free 3′-hydroxy termini generated by DNA breaks and caused higher γH2AX levels. Both levels were then further increased following HDAC inhibitors application. Changes at the molecular level were noted in the expression and activity of DNA repair genes and their protein products. For example, Rad51 is an important factor in homologous recombination, and while this was down-regulated in PAM treated cells, the effect was markedly greater when PAM application was combined with HDAC inhibitors. Although PAM alone slightly reduced the expression of Ku70 and Ku80 involved in non-homologous recombination, this was again more significantly decreased with simultaneous application of HDAC inhibitors. The prior demonstration that down-regulated HR and non-homologous end joining pathways resulting in decreased DNA repair ability after HDAC application is also interesting and important [165]. In addition, poly ADP-Ribose Polymerase-1 (PARP-1) was activated in these cells as a result of DNA fragmentation. PARP-1 activation is a noted early response in DNA damage detection and repair, and it provides an essential component in base-excision repair (BER) [166]. PARP activation occurred after cells were exposed to PAM, but this effect was again enhanced by the application of HDAC inhibitors. The authors of this study consider that the intensive PARP activation leads to cell energy-source depletion because this was accompanied by high NAD+ consumption and abnormal ATP depletion. In addition, PARP-1 can be degraded by poly ADP-ribose glycohydrolase to monomeric ADP-ribose which is a primary activator of transient receptor potential-melastatin 2 (TRPM2) [167]. The increased TRPM2 activity was previously reported to elevate [Ca2+]i through the influx of extracellular Ca2+ and release of lysosomal Ca2+ [167]. Any imbalance in this release adversely affects cells and can often leads to autophagy [168].

There was also increased PARP1 activity in A549 cell lines from higher oxidative stress following CAP exposure [169]. However, this was only associated with DNA repair, and the targeted inhibition of PARP augmented apoptosis. It is necessary to further examine PARP expression and activity, especially if there is simultaneous application of other agents, such as HDAC inhibitors [170]. In addition, the increased PARPI activity under certain circumstances can also be associated with induced cell death [171,172]. In summary, HDACI’s role in association with CAP exposure can be very important, but it requires further analysis because (1) CAP’s effect was noted together with simultaneous HDAC increase [163], and (2) the most significant and enhanced effect was achieved with HDAC inhibitors when PAM was applied to the cell lines [164]. The effects of CAP application on epigenetic changes are listed in Table 1.

## 6. CAP and Non-Coding RNAs

The expression and activity of miR-19a-3p changes after CAP application were demonstrated in MCF-7 BC cell lines [173]. This miRNA is considered oncomiR which is abnormally highly expressed in gastric and pancreatic cancers, and it is associated with poor prognosis [174,175]. The authors transfected MCF7 cells with mir-19a mimics and found that their proliferation increased 2.5-fold. However, CAP application for 10 × 30 s and 10-min significantly inhibited this proliferation. Both doses had approximately the same effect on anti-proliferation and reducing the miRNA’s expression. The authors of this study considered that this should be due to hypermethylation of its promoter region, with significant correlation between expression and methylation rate. In addition, the *ABCA1*, *HBP1, GJA1*, and *PTEN* genes have been demonstrated to be target genes for this miRNA. The *HBP1,* GJA1 and *ABCA1* genes are associated with both the onset and development of BC [176,177,178], and abnormalities in the activity of the important *PTEN* tumour suppressor have been linked to similar outcomes [179]. The application of ROS inhibitors then reduced CAP influence on this miRNA’s expression and cell proliferation. Finally, the research recorded that CAP affected the methylation of the miR-19a, miR-1723, and miR-18a promoters and decreased their expression. The results, however, lacked statistical significance. 

CAP exposure can also affect lncRNAs’ activity and expression. The authors Kim et al. demonstrated that CAP can influence the expression of multiple genes dependent on exposure time [180]. The most significant change in expression occurred in the *ZNRD1* gene. Although uninterrupted 10-min exposure increased this gene’s expression, repeated shorter exposure for 10 × 30 s reduced its expression. The ZNRD1-AS1 lcnRNA is encoded by its antisense strand, and it is important that although CAP exposure for 10 × 30 s increased its expression, the 10-min exposure decreased its expression. Therefore, the CAP effect on this lncRNA expression was opposite to its effect on *ZNRD1* gene expression. ZNDR-1AS subsequently affected the activity of its *HLA-A, HCG9*, and *PPP1R11* cis-genes. A previous study demonstrated that CpG sites near the *ZNRD1* promoter are hypermethylated in BC cell lines, and that CAP application can elevate this level [155]. Moreover, the antisense-strand coding this lncRNA shares the CpG site [180].

It is somewhat surprising that although different CAP exposure times caused diverse *ZNRD1* gene expression, both doses elevated CpG methylation levels in its promoter region. The 10-min exposure induced 96% methylation level increase, and the 10 × 30-s exposure increased it by 38%. This indicates that the CpG sites does not affect the expression of ZNRD1 and ZNRD1-AS1, although CAP alters their methylation level. The authors considered that the most likely explanation for this is that ZNRD1 induces ZNRD1-AS1 down-regulation. This was confirmed by ZNRD1 inhibition by siRNA which induced ZNRD1-AS1 up-regulation, but ZNRD1-AS1 targeting by siRNA did not affect *ZNRD1* expression. The CAP application effects on changes in ncRNA’s expression are listed in Table 1.

**Table 1:** List of CAP effects on epigenetic changes and non-coding RNA expression and action.

Type of Epigenetic Change	Plasma Type/Gas Injected	Resulting CAP Exposure Effect	Exposure Time	Method Used for Analysing Epigenetic Status	Type of Cells/Tissue Analysed
DNA methylation [155]	DBD plasma device	Low decrease in *Alu* sequences methylation in MDA-MB-231 cell lines, wide DNA methylation profile changes detected with microarray analysis—318 hypermethylated CpGs and 56 hypomethylated CpGs in MCF-7 cells, 76 hypermethylated CpGs, and 63 hypomethylated CpGs in MDA-MB-231 cells	30 s/10 times	Pyrosequencing, microarray	Breast cancer cell lines MDA-MB-231 and MCF-7
DNA methylation (presumably), Histone acetylation (presumably) [157]	DBD plasma device, Helium injected 5 min before the experiment into the reaction chamber	Changes in methyltransferase activity after 3-min CAP exposure; increased expression of genes involved in histone acetylation after 3-min CAP exposure	(a) 1 min, (b) 3 min; cells harvested 1,2,4,8 hours after exposure	Microarray, validation with qPCR	Lung-cancer cell lines A549
Histone methylation [160]	DBD plasma device	899 sequences within promoter regions showed changes in H3K4me3 methylation level after CAP application; statistically significant correlation of several genes’ expression with histone methylation changes	10 min	Genome-wide ChIP-seq, qPCR	Breast cancer cell lines MCF-7
Histone acetylation [163]	DBD device fed with Helium	Increase in Histone deacetyl-transferase 1 activity and decrease in Histone 3 acetylation	50 s every hour/10 times	qPCR, Western blot, RNASeq	Adipose tissue-derived stem cells
miR-19a-3p expression reduction [173]	Mesh-DBD	Decrease in miR-19a-3p expression due to hypermethylation of its promoter, changed activity of miR-19a-3p subsequently affected its downstream genes	(a) 30 s every hour/10 times; (b) 100 s; (c) 600 s	qPCR, microarray	Breast cancer cell lines MCF7, MDA-MB-231
ZNRD1-1AS1 lncRNA expression changes [180]	mesh-DBD	Expression of *ZNRD1* gene is affected by CAP and it presumably regulates expression of its antisense lncRNA ZNRD1-1AS1, subsequently ZNRD1-1AS1 lncRNA regulates its cis-genes	(a) 30 s/10 times; (b) 600 s	qPCR, methylation specific-PCR	Breast cancer cell lines MCF-7

CAP—Cold atmospheric plasma, ChIp-Seq—Chromatin immunoprecipitation sequencing, DBD—Dielectric barrier discharge, lncRNA—Long non-coding RNA, min – minute; miR—microRNA qPCR—Quantitative polymerase chain reaction, RNAseq—RNA sequencing; s – second.

## 7. CAP as a Tool for Synergistic Cancer Therapy

Current medical anti-cancer treatments often have side effects because of the necessity to administer higher and repeated doses to achieve therapeutic effect [181]. Therefore, the main aim of current global research is to achieve administration of lower pharmaceutic doses with the same final effect on patients. The following research shows that CAP has the potential to enhance effectiveness in synergistic treatment with pharmaceuticals and chemotherapeutic agents.

### 7.1. Cisplatin

Herein, oral squamous cell carcinoma cell lines SCC-15 and control human gingival fibroblast lines HGF-1 were exposed to individual CAP and cisplatin treatment, and then their combined application [182]. Sole cisplatin treatment decreased the viability of both tumorous and control cell lines in a dose-dependent manner, but the decrease in tumour-cell viability was more significant when combined with CAP. Moreover, the decrease in fibroblast cell viability was significantly lower in all sole cisplatin and CAP treatments, and in their combined application compared to the SCC-15 tumour cell lines. The treatment regime with 3μM cisplatin application caused loss of viability in 50% of tumour cells, but only 10% decrease in fibroblast viability. Sole CAP treatment was dose and time dependent—3-min exposure enabled only about 50% of tumorous cells to remain viable, but over 70% of fibroblasts remained viable. The combination of 1μM cisplatin with 3-min CAP treatment or 3μM cisplatin and 1-min CAP treatment were determined optimal for the desired synergistic effect on SCC-15 cells. These also had no significant negative effect on the control fibroblasts. In comparison, 1μM of cisplatin without CAP effected lost viability in only 25% of SCC-15 cells, but its combination with 3-min CAP caused 60% loss of cell viability. 3μM cisplatin without CAP application then caused 50% SCC-15 cell lost viability, and its combination with 1-min CAP exposure increased this to 60% cell loss. Therefore, CAP enhanced cisplatin effect; at least under in vitro conditions. In addition, the Ic50 cisplatin dose was achieved in HGF cells at a 5.7-fold higher dosage than that required for SCC-15 cells. The precise figures here were 16μM compared to 2.8μM. Finally, the CAP application time needed for fibroblast Ic50 was almost twice as long as that required for SCC-15 cells at 300 s, compared to 173 s.

Although cisplatin has been used as a routine therapy in many cancers for several decades, its use has undesirable side effects. These include electrolyte and auditory disorders, bone marrow suppression, and nephro- and cardiotoxicity [183]. CAP application may therefore be considered a potential mechanism of enhancing cisplatin efficacy and provide both reduced dosage and side effects. The combined administration of CAP and cisplatin had a more pronounced effect on the expression of some genes involved in apoptosis compared to that achieved by sole cisplatin or CAP application. These genes include *PTEN,* and those coding Caspase9 and p53. This effect was more pronounced in cancer cells than in fibroblasts [182].

### 7.2. Sulfasazaline

Sulfasalazine (SSZ) is an anti-inflammatory used in the treatment of rheumatoid arthritis**,** inflammatory bowel disease and chronic retinal inflammation [184]. This SSZ anti-cancer efficacy has been demonstrated to increase the oxidative stress and cause cysteine deficiency in tumour cells [185]. Cysteine is an important precursor in the synthesis of intracellular-reduced glutathione (GSH). Abnormal GSH status in cancer cells is associated with increased cell growth, invasion, and chemotherapy resistance. In contrast, its reduced synthesis has also been demonstrated in induced cancer cell death [186].

The analysis of combined applications of SSZ with CAP and SSZ with X-radiation (XR) in colorectal carcinoma HCT-116 cell lines and human T lymphoblast Molt-4 cell lines has demonstrated important associations which can be exploited to improve anti-cancer strategies using CAP devices [187]. The sole application of SSZ in that analysis had only minimal effect on DNA fragmentation, apoptosis rate, and cell survival. However, the application of CAP or XR had similar effect in markedly increasing DNA fragmentation and apoptosis rate. In addition, a greater percentage of cells undergo early apoptosis after both CAP and XR exposure, with a lesser percentage experiencing secondary necrosis. Moreover, their combined application with 0.25 mM SSZ significantly increased DNA fragmentation and apoptosis rate, compared to their sole application. The applications of SSZ with CAP and SSZ with XR had similar final effect on both DNA fragmentation and apoptosis rate, and a significant reduction in intracellular GSH levels. Therefore, the resultant influence of both CAP and XR combined with SSZ were similar in their final effects. However, XR application induced known negative side effects, while CAP treatment had none, or at least none which were significant. Therefore, CAP can be considered a safer alternative to XR.

Although CAP and XR have similar final effects, differences are noted at the molecular level. This particularly involves the significant increase in ROS levels following CAP application, regardless of whether it is combined with SSZ. In particular, 60-s CAP exposure produced 24-times more ROS than a 2Gy XR dose. However, XR significantly increased Ca^2+^ to greater cell levels than CAP. Combined CAP and SSZ enhanced apoptosis through the intrinsic apoptotic pathway. This was demonstrated with decreased expression of anti-apoptotic Bcl-2 and Bcl-xL and increased caspase-3 protein level. Combined IR/SSZ application also decreased anti-apoptotic proteins and increased caspase3, but this was accompanied by the increase in the levels of FAS, which is a typical cell membrane death-receptor associated with extrinsic pathways. In addition, FAS was not increased when XR was applied alone, so this increase occurs only with XR and SSZ combination. Finally, it was possible to minimise all IR and CAP effects by applying N-acetyl cysteine.

### 7.3. Tamoxifen

Tamoxifen is routinely used to treat BC, and it is estimated that its application in ER-positive BC reduces the overall mortality rate by up to 30%. However, more than half of ER-negative BCs are intrinsically resistant to tamoxifen, and a high percentage of patients acquire resistance during treatment [188]. Therefore, future goals include increasing its efficacy, especially in ER-negative BC, by eliminating patient resistance. This can be achieved by tamoxifen combination with agents which can diversely affect the expression of target genes and signalling pathways.

CAP’s selective effect on gene expression and cell signalling has also been demonstrated, particularly in tumour cells, and this can potentially reverse drug resistance [19,189,190]. Tamoxifen-resistant MCF-7 cell lines were specially developed to verify if CAP can affect this resistance [191]. CAP application for 10 × 30 s restored tamoxifen sensitivity in these resistant cell lines by up to 50%. Genome-wide expression analysis then demonstrated significant change in the expression of 20 genes connected with tamoxifen resistance in the MCF7 cell. Moreover, the expression levels of 18 of these genes had opposing patterns after CAP exposure. CAP application decreased gene expression when the genes in tamoxifen-resistant MCF7 cells were initially up-regulated, and CAP increased the expression of initially down-regulated genes. Therefore, CAP exposure reversed the expression levels of 18 of these 20 genes to levels almost identical to those in standard MCF7 cells.

The authors then recorded that two of these 18 genes mediated restored sensitivity to tamoxifen. These were the *MX1* and *HOCX6* genes, and their expression reverted to the levels of normal MCF7 cells after CAP treatment. This reversion involved both the genes’ mRNA and protein levels [191]. All the above results highlight CAP’s potential as support-therapy for patients with tamoxifen-resistant BC.

### 7.4. Doxorubicin and Epirubucin

Sagwal et al. studied the effects of CAP and various cytotoxic agents on several mice and human melanoma cell lines [192]. They focused on malignant melanoma because it is often resistant to chemotherapy, and it consequently has poor prognosis. The first step was sole application of the doxorubicin, epirubicin, oxilaplatin and vorinostat cytotoxic agents, and these caused DNA damage and micro-nuclei formation. This step was followed by the simultaneous application of a cytotoxic agent and 30-s CAP exposure. This combination produced significantly increased cell DNA damage and higher H2AX and ATM phosphorylation. Most importantly, the cytotoxic effect of doxorubicin or epirubucin application was achieved at sub-micromolar dosage, with 10-fold lower concentration than the standard Ic50. In addition, there was no increased micro-nuclei formation in sole CAP application, and CAP also up-regulated the SLC22A16 cationic transporter levels at the molecular level. This transporter was previously described as a doxorubicin importer [193]. In summary, synergistic CAP application increased doxorubicin and epirubicin cytotoxicity and oxaliplatin’s additive effect on melanoma cells. Finally, some authors observed the concomitant increase in the secretion of ATP and CXCL10 immunogenic cell-death markers in some melanoma cells after this combined treatment.

### 7.5. Decarbazine

Decarbazine (DAC) chemotherapy is also used to treat melanomas. As with other chemotherapeutic agents, DAC has side effects which should be minimised Here, the combination of DAC and CAP appears promising; at least under experimental in vitro conditions [194]. The effects of separate CAP and combined CAP/DAC application on mouse B16 tumour and L929 fibroblast cell lines have been analysed. CAP alone significantly affected cell growth, apoptosis, and changes in the expression of genes associated with autophagy in B16 lines, but its effect was only minimal in fibroblasts. Stimulation of autophagic cell death is the goal of many researchers, because they consider it a promising alternative method of killing melanoma cells which are largely apoptosis-resistant. [195]. Sole application of DAC was less selective than CAP, and it induced apoptosis and caused similar reduced viability in both tumour and non-tumour cell lines under in vitro conditions. However, it is important that the final effect of the DAC/CAP combination on cell viability and molecular activity was comparable to DAC monotherapy, and was also non-selective. Individual therapy with CAP or DAC under in vivo conditions led to tumour mass shrinkage in the mouse B16 xenografts, but the most significant tumour reduction was observed when combined therapy was applied. In addition, the expression ratio of apoptotic and anti-apoptotic genes in the mouse models varied significantly in monotherapies and the CAP/DAC combination.

Treatment herein was divided into three stages lasting five, ten and fifteen days; (1) CAP alone had no significant effect on Bax/Bcl2 ratio or caspase 3 expressions after the fifth experimental day, but sole DAC therapy increased the levels of Bcl2 and caspase 3. Combined CAP/DAC therapy then increased the levels of both Bax and Bcl without ratio change, and increased Caspase-3 expression; (2) sole CAP and DAC applications and combined CAP/DAC therapy all increased Bax/Bcl2 ratio and caspase 3 expression after the tenth day; (3) the two individual treatments and CAP/DAC combination did not affect the Bax/Bcl2 ratio after the 15th day, but they significantly increased caspase-3.

CAP treatment also enhanced the overall expression of *LC3* and *ATG3* autophagy genes compared to DAC treatment. However, this expression was even more enhanced after synergistic treatment. This was particularly noticeable in the combined therapy after day 5, when *LC3* was expressed 20-fold and *ATG3* 40-fold times the untreated expression rates.

### 7.6. CAP and Plasma-Activated Infusion

CAP application is currently largely limited to surface structures, so research is now endeavouring to extend CAP effects to subdermal and internal structures to affect internally deposited tumours and to treat other internal diseases. This can be achieved by designing a sufficiently miniature and effective needle-like CAP device able to penetrate close to sub-cutaneous and internal structures, and endoscope-like CAP device. An alternative is using PAL and PAW to flush the oral cavity and access internal structures by suitable approaches such as plasma-activated infusions [PAI] [44,45,46,47,48].

This PAI was prepared by Argon CAP irradiation [196], and it has selectively decreased melanoma and osteosarcoma cell-line viability in in vitro conditions; but not for dermal and lung fibroblasts. Here, the PAI effect could be reversed by MnTBaP and N-acetylcysteine antioxidants. In addition, salinomycin (SAL) is a polyether that acts as a potassium and calcium ionophore [197]. Its combined use with PAI in mouse osteosarcoma allografts had a much greater effect on both reducing tumour mass and metastasis than either sole therapy. PAI also stimulated the induction of necroptosis at the molecular level. This was mainly caused by RIP1 and RIP3 kinase activation, while the cyto-protective autophagy herein was suppressed by mTORC 1/2 activation. In addition, the ROS present in PAI significantly disrupts mitochondrial networks. PAI also increased Raptor, Rictor and p70-S6 kinase phosphorylation and decreased LC3-I/II expression. The combined application of PAI and SAL has the greatest effect on mitochondrial networks and homeostasis. However, changes in other cell molecular mechanisms following the PAL/SAL application which led to this non-apoptotic cell death remain unknown. Finally, no significant side effects were observed in mouse models when this combination was applied, and while this may be a potential treatment for apoptosis-resistant cancers, it requires further analysis.

### 7.7. Temozolomide

CAP application amplified the temozolomide (TMZ) cytotoxic effect in U87MG glioblastoma cell lines, and this CAP/TMZ combination caused greater viability loss than TMZ alone. This was also associated with higher histone H2AX phosphorylation [198]. It is accepted that the normal response to TMZ application is increased activity of O6-methylguanine-DNA methyltransferase (MGMT). This is a reputed “suicide” DNA repair enzyme [199]. Although glioblastomas generally have high expression of this gene which makes them largely resistant to the effect of TMZ, some of these cell lines have reduced gene expression. This is usually due to promoter methylation, and these have notably higher sensitivity to TMZ [200]. However, CAP application enhanced this cytotoxic effect, even in cell lines with highly expressed MGMT [201], and this cytotoxic effect was comparable with that in cell lines with methylated MGMT. Finally, synergistic application of CAP and TMZ leads to higher frequency of G2/M arrest than the individual therapies, and subsequent apoptosis. However, the precise changes in MGMT promoter methylation after CAP exposure remain unclear, and this requires elucidation.

### 7.8. CAP and Photodynamic Therapy

CAP combination with photodynamic therapy also appears promising in cancer treatment [202]. Photodynamic therapy (PTD) employs a photo-sensitiser (photosensitive drug) activated by light from different sources, including laser and LED lighting. The photo-sensitiser is first distributed throughout the tumour and then is activated by emitted light, reacts with oxygen so that singlet oxygen is generated [203]. CAP and phototherapy using 5-Amino-Levulinic acid (ALA) was tested on A549 human lung-carcinoma cell lines [202]. The cells were subjected to combinations of individual CAP and ALA application and PTD, ALA/CAP and PTD/CAP treatments. The author’s design for simultaneous CAP/PTD application was interesting because it comprised a plasma jet nozzle surrounded by LEDs at appropriate angle so that both plasma jet and LED light focused on the same point. The cells were exposed to various exposure times and treatment combinations. The 30-s exposure provided a combined CAP/PTD effect on cell viability 18% higher than sole PTD, and 23% higher than CAP alone. The 60-s combined CAP/PDT exposure then decreased cell viability by 37% more than individual PTD and 41% more than CAP application. However, maximum enhancement remained at approximately 40% in longer exposure, and did not further increase the combined PDT/CAP effect over CAP or PTD sole therapies. These results indicate that combined CAP and phototherapy could be effective, especially in the treatment of accessible tumours such as skin cancer.

### 7.9. Synergistic Effect of CAP and Nanoparticles

CAP potential should not be limited to synergistic treatment with routinely used pharmaceutical agents because it could also enhance nanoparticle therapies. Nanoparticle systems are currently being developed to reduce the adverse effects of standard chemo- and radiotherapeutics. The use of these nanoparticle systems can be beneficial in improving hydrophobic drug delivery and in reducing metabolic drug degradation and targeting specific cells [204,205].

Kim et al. instituted a pilot study where G361 melanoma cell lines were exposed to the following; CAP, CAP/gold nanoparticles (GNP) without antibodies and CAP/GNP bound to anti-phospho-focal adhesion kinase (FAK) antibodies [206]. The FAK antibodies were chosen, because high *FAK* gene expression is a marker in many cancer types [207,208]. Although CAP alone increased these cells’ death rate by 14%, combined CAP/GNP reduced it by 36% and combined CAP/FAK/GNP reduced it by 74%. Therefore, this last combination increased the death rate approximately 5-fold compared to CAP alone.

Zhu et al. applied 5-FU encapsulated electro-sprayed core-shell PLGA nanoparticles to the MDA-MB-231 metastatic BC cell line, then CAP alone and finally CAP/PLGA nanoparticles [209]. Although the individual nanoparticles and CAP reduced cell viability, the most significant reduction was achieved by synergistic CAP/nanoparticle application. In addition, while CAP facilitated nanoparticle cell penetration and diminished microvilli and pseudopodia, the precise molecular mechanism of this facilitation was not defined in the study. Finally, CAP treatment also induced down-regulated expression in the *VEGF*, *MTDH*, and the two *MMP2* and *MMP9* matrix metallo-proteinase genes.

GNP synergistically applied with CAP can increase U87 glioblastoma cell death-rate by up to 30% compared to CAP alone [210]. However, the resultant effect in that study largely depended on correct GNP concentration. In addition, Elgendy and Abdallah analysed CAP discharge and GNP physical interaction [211]. The CAP and nanoparticle surface interaction provided further production of singlet oxygen groups. These were especially ^1^O_2_, triplet oxygen ^3^O_2,_ and the OH groups. The GNP therefore enhanced CAP effect by producing these additional singlet oxygen molecules and OH groups. Although the final oxidative stress effect can be associated with ROS concentration, the particles’ actual performance as solid and stable ROS generators in complex living systems requires further investigation.

In addition, He et al. [212] consider that CAP enhances cell uptake and accumulation of these nanoparticles. Their investigation was performed on U373MG glioblastoma multiforme cells which could accumulate significantly more AU nanoparticles after 30-s exposure to CAP generated by DBD. This higher ability was observed one and two days after exposure. In addition, the authors observed physical and morphological cell membrane changes in the U373MG cell lines. However, most of these alterations were only temporary. The authors then hypothesised that CAP treatment caused membrane damage which then triggered membrane repair response. This involved rapid endocytosis to remove damage parts and increased membrane permeability to return cell integrity. This resulted in increased cell Au nanoparticle uptake during repair, and subsequent increased cytotoxicity caused by these nanoparticles. These processes are supported by the Au nanoparticles found preferentially in lysosomes and other acidic vesicles. In addition, Recek et al. also analysed CAP effects on tumorous and healthy cell membranes [213]. They recorded that CAP caused significant and irreversible morphological and functional changes in cancerous U87 cell lines, and only minimal and recoverable changes in the control E6/E7 bronchial epithelium cell lines. Most importantly, the authors discovered that CAP caused temporary disappearance of microvilli in control cells but had no effect on the U87 cancerous cell-line’s invadopodia. Finally, He et al. demonstrated that the cytotoxic effect was 25 times higher in U373MG cell lines after simultaneous application of CAP and Au nanoparticles than in sole Au-particle treatment.

Other researchers reported that combined CAP and iron-oxide-based magnetic nanoparticles (MNP) can achieve the similar cytotoxic effect as GNP [214]. The CAP/MNP combination decreased A549 cell-line viability more significantly than all individual MNP concentrations and CAP exposure times. These specific conditions were 0.0035; 0.035; 0.07; 0.14 and 0.28 mg/mL concentrations at 0, 30, 60, 90, and 120 s, respectively. The only exception was 150-s CAP application which decreased viability comparable to the CAP/MNP combination. Intracellular reactive species levels were also higher in the CAP/MNP synergistic group than in individual treatments, and this synergy exerted the strongest inhibition on cell migration. This result enabled the authors to predict that epithelial-to-mesenchymal transition can be widely affected by this synergistic treatment. This prediction was confirmed by the results that E-cadherin was most significantly up-regulated in treated cells and the vimentin was most significantly suppressed. These authors then analysed EGFR expression in individual CAP application. EGFR over-expression is one of the most typical markers of lung cancers [215,216], and CAP reduced the dose-dependent mRNA and protein levels. It is interesting that dual CAP-MNP treatment did not further decrease this expression with statistical significance. CAP alone also decreased phosphorylation levels of the EGFR’s downstream kinases AKT and ERK, and while this was increased by the dual therapy, individual MNP application had only a mild effect on the phosphorylation changes.

The authors then injected A549 cell lines into mouse xenografts. Combined CAP/MNP treatment resulted in approximately 60% reduction in tumour size, significantly more than CAP alone. It is important here that tumour volumes were only slightly less reduced following sole MNP application compared to the CAP/MNP treatment, and that the dual therapy significantly condensed tumour cells nuclei. This condensation indicated intensive necrosis. Moreover, TUNEL assay revealed a higher percentage of apoptotic cells in tumour tissue after CAP-MNP treatment. This dual treatment therefore most likely induced more cell-death types in the mouse xenografts. Finally, the E-cadherin expression level was markedly increased in these xenografts following combined treatment, and vimentin expression was most significantly reduced. These results were similar to the occurrence in cell lines. Finally, the Ki67 proliferation markers levels were also lowest after dual therapy.

### 7.10. CAP and Lysozyme

Synergistic CAP use with enzyme lysozyme presents a very promising possibility. Lysozyme is produced as a non-specific defence mechanism, and it is associated with the monocyte-macrophage system [217]. It has been also shown that lysozyme has anti-cancer activity and can directly activate immune cells or increase tumour-cell immunogenicity [217]. In addition, CAP can influence lysozyme structural and functional changes [218]. Choi et al. [218] exposed lysozyme to 8-min and 12-min CAP exposure. They employed both DBD and the APPJ plasma jet. Nitrogen and air were used as feed gases for plasma generation. Important differences were noted in secondary protein structure with DBD and plasma jet usage. The α-helix percentage decreased with DBD, and increased with APPJ use. This difference was more significant with nitrogen usage than with air. Moreover, X-ray chromatography indicated that both devices influenced structural changes in both Loop3 and Loop6. Although CAP application also affected the W62, W108, and D101 substrate binding sites, this only occurred after 12-min CAP exposure. It was then further demonstrated that both CAP devices decreased lysozyme activity, but the APPJ and nitrogen use caused the greatest loss.

CAP application also affected lysozyme thermodynamics crucial for its correct function. This application decreased the temperature at which 50% of lysozymes are unfolded. Takai et al. [219] also observed altered lysozyme structure and decreased enzymatic action after CAP application. They suggested that these changes were due to ROS and RNS action, rather than to UV radiation and resultant plasma heat. Choi et al. [217] followed their previous analysis [218] with the consideration that special conditions must be met for appropriate CAP effect on lysozyme structure and activity [217]. Experimental pH was a very important condition. All lysozyme solutions were prepared in 10mM phosphate buffer at pH 2 and pH 7 and then treated similarly. The DBD device was used for 5, 10, and 15 min with 5mm distance. Different lysozyme conformation and thermal stability were noted at different pH. The CAP treatment at pH 2 resulted in increased oxidation and complete loss of lysozyme activity. In contrast, the experimental conditions at pH 7 increased lysozyme activity to 118% after 5-min CAP treatment, and to 125% after 10 min. The 15-min treatment, however decreased the activity to 80% of the untreated controls.

Finally, lysozyme exposed to CAP at pH 7 for 5 and 10 min significantly decreased viability in the K-MEL2 and G361 cell lines compared to untreated lysozyme. It also increased caspase-3/7 activity, and caspase-9 activity to a lesser extent in tumour cells. Caspase activity in the control HaCat cells remained unchanged after lysozyme application. Although these results suggest that the lysozyme with modified structure and activity application has potential use in cancer treatment, these are pilot studies, and the effects in in vivo conditions must be further analysed**.** The effects of CAP application in synergistic treatment are listed in Table 2.

## 8. Cold Atmospheric Plasma, H2AX Phosphorylation, DNA Damage, and Apoptosis

Increased H2AX histone phosphorylation is a typical sign of DNA damage, and especially for double-strand breaks (DBS). This core histone protein variant is phosphorylated and localises DNA damage sites shortly after the DBS. In addition to the association between H2AX phosphorylation and DNA damage, significant association of this phosphorylation and apoptosis has also been demonstrated [122,123,124]. However, the association of phosphorylation, DNA damage, and apoptosis is quite complex, and there are two possibilities; (1) if the DNA damage is too extensive and therefore irreparable, it can lead to apoptosis and, (2) apoptosis can be induced by a various internal and external triggers and this can finally produce DNA fragmentation accompanied by increased H2AX phosphorylation [115,220,221,222] In addition, DBS can also be caused by free oxygen radical action [223].

Arndt et al. [224], found that 2-min lasting CAP exposure caused DNA damage in various melanoma cell lines, promoted Sub-G1 phase induction and strongly increased apoptosis with a high degree of Cytochrome C release and caspase3 activation. In contrast, 1-min contact caused significantly lower apoptotic rate, but this was combined with signs of cell senescence and especially a very high H3K9 trimethylation rate.

Further research revealed high H2AX histone phosphorylation after only 1-s CAP contact with oral cavity squamous carcinoma cell line [225]. In addition, Comet assay showed overall higher DNA damage. This damage was accompanied with increased p21, AKT and p53 expression, and decreased cyclin D1 phosphorylation. These changes caused sub-G1 cell-cycle arrest and subsequent p53/AKT mediated apoptosis. Apoptosis did not occur in cell lines with mutated p53, and inhibited cell-cycle progression was partly over-ridden by suppressed ATM expression due to its specific siRNA.

Cell cultivation in PAM also led to H2AX histone phosphorylation [226] These experiments were performed on HCT116 colorectal multi-cellular tumour spheroids. The medium was initially exposed to CAP for 120 s and the spheroids were then transferred into this medium after 1, 14, 24, and 48 h. Spheroids transferred up to 14 h after exposure had approximately 40% reduced growth and then decreased to approximately 20% after 24 and 48 h, while the greatest H2AX phosphorylation was observed after 1 h and then continued to decrease. The authors then exposed the spheroids to PAM for 60 and 240 s. The 240-s exposure caused the most significant change in H2AX histone phosphorylation, and its absolute extent was dose-dependent, and the phosphorylation here was mainly detected in the outer-most layers. However, the DNA damage and H2AX phosphorylation were reversible with catalase induction, and 30% decrease in DNA damage was observed following application of super-oxide dismutase and D-mannitol scavengers which act against •O_2_^–^ and OH•. In addition, there was also decreased viability, increased DNA damage and H2AX phosphorylation following H_2_O_2_ addition to the culture medium without prior plasma exposure.

Kaushik et al. [227] compared the effect of CAP and chemically induced ROS systems on various cell lines. These systems included: (1) xanthine with xanthine oxidase, which primarily generate O_2_^−^; (2) HO· radicals formed in the Fenton reaction, (3) H_2_O_2_ which was directly added to the cell culture from stock solutions. CAP and chemical agents have been applied to the following cell lines—T98G glioblastoma lines, A549 lung-cancer lines, HEK293 lines derived from human embryonic kidney and MRC5 fibroblast lines. CAP application was found to selectively affect viability only in tumour cell lines and not in healthy cell lines. The apoptosis rate was significantly increased in tumour lines by the caspase mechanism associated with altered expression in both pro-apoptotic and anti-apoptotic genes. Moreover, H2AX histone expression and degree of phosphorylation were both increased.

CAP application also altered phosphorylated ERK1/2/MAPK protein levels, but this plasma effect was mostly reversible with ROS scavengers. In addition, chemical systems non-selectively decreased viability in both cancerous and non-cancerous cells, and the reversal of this effect with ROS scavengers was not as pronounced as in CAP. However, it is important that CAP increased caspase 3/7 expression in healthy cells by 180–200%, although it did not lead to adequately increased apoptosis rate. Finally, the authors considered that caspase activity could have additional cell functions unrelated to apoptosis.

Plewa et al. [228] then investigated plasma’s effect on DNA damage in 3-dimensional HCT116 spheroid models. The highest phosphorylation was again observed in the outer-most cell layers. Although Plewa et al. [228] used direct exposure to the CAP plasma pen instead of PAM, these results were similar to those in Judee et al.’s experiments [226]. The highest H2AX phosphorylation immune-histochemical signal was observed 4 h after treatment, but decreased significantly after 16 h and completely disappeared in 24 h. The authors also recorded that the outer-most layers affected by CAP significantly disintegrated and perished. The degree of disintegration was directly proportional to CAP’s effect and the degree of H2AX phosphorylation. Surprisingly, they did not detect apoptotic cells with PARP-C staining, and they most likely presumed that the observed decline in growth was associated with inhibited cell proliferation and not detectable apoptosis. Moreover, the rate of DNA damage was significantly reduced after application of antioxidant N-acetyl cysteine which is a free radical scavenger, and no H2AX phosphorylation was also detected following this application. Moreover, the cell-line growth inhibition correlated also with Ki67 marker loss. Although the particular cell-death types and processes were not described in this study, the ROS produced by CAP significantly contributed to reduced viability.

The above studies therefore suggest that CAP exposure can lead to the following possibilities: (a) tumour-cell DNA damage is directly caused by high levels of the ROS produced by CAP, and this damage subsequently leads to apoptosis or other types of cell death; or (b) CAP triggers apoptosis, and this then led to DNA fragmentation and higher levels of H2AX phosphorylation. However, the CAP-produced-ROS have an important function in both scenarios. ROS can penetrate the cancer cell more easily due to these cells’ different membrane structure, and especially their membrane lipid composition [70]. In addition, the extracellular matrix in tumour cells naturally has higher amounts of •O_2_^−^ super-oxide [229]. Although several additional studies have directly associated CAP exposure with the formation of DNA breaks [230,231,232], Bekeschus et al. provide a different perspective [122]. These authors assume that the H2AX histone phosphorylation results from triggering apoptotic cascades rather than from DBS directly caused by ROS. This is based on this histone’s phosphorylation with pleiotropic cell functions, and apoptosis is also associated with its increased phosphorylation. [122,123,124] and the phosphorylation of this histone is ten-fold higher in apoptotic than non-apoptotic cells [125].

The authors also based their statement on the following (1) ROS are not produced inside the cell, but in the external environment where they have a relatively short lifetime, and only some of the initially generated ROS actually arrives in the cells [233,234], (2) there are several ROS scavengers in the cell cytosol [235]; and (3) ROS must be able to penetrate the endoplasmic reticulum and nuclear membranes after overcoming the cell membrane, and they are exposed to other antioxidant such as PRDX2 in those environments [236].

The question therefore is “can the ROS produced by CAP enter the nucleus in sufficient amount and remain sufficiently reactive to directly damage DNA?”. In answer, the authors performed the following experiments. The TK6 lymphocyte cell lines were exposed to the following three ROS inductors: CAP, H_2_O_2_, and hypochlorous acid. The experimental control was exposure to UV-B light. The cytotoxicity of all factors except UV-B could be reversed by antioxidants, and the degree of H2X phosphorylation was not affected by cell-cycle phase. The proliferating cells in the S or G2 phase were exposed to CAP, and the resultant ratio of phosphorylated H2AX was even lower than in the untreated control cells in the G1 phase. This is interesting, because DNA is unwound in the proliferating cells in S and G2 phase, and this makes DNA more susceptible to ROS damage.

Herein, only UV-B light caused formation of micro-nuclei which are considered the functional read-out of genotoxic DNA DSB. In addition, the application of both the MAPK-p38 and pan-caspase inhibitors significantly reduced H2AX phosphorylation rate following CAP and other ROS inductors application but not after exposure to UV light. In addition, the application of the ATM inhibitor also reduced phosphorylation rate, but this caused only statistically insignificant differences. These results suggest that plasma-induced γH2AX phosphorylation more likely depends on stress and apoptosis signalling pathways than on DNA damage caused by direct ROS penetration to the nucleus.

Therefore, the H2AX induced by CAP exposure can be considered a marker of oxidative stress rather than DNA damage. In addition, other studies suggest that γH2AX also has an important function in antioxidant defence signalling [237], because H2AX knock-out increases endogenous ROS levels. This knock-out also caused failure to activate antioxidant response elements through nuclear E2-related factor 2(Nrf2) [238], and subsequent mitochondrial damage [239]. However, further research is required to elucidate if CAP can directly cause DNA damage, and the investigation could focus on short-term kinetic measurements of the γH2AX-level according to the onset of apoptosis. Further benefits could evolve from analysing the activity of the *Nbs1*, *53BP1*, and *Brca1* genes coding DNA repair-associated proteins. Higher activity of these genes and their proteins could also mark double-strand breaks and H2AX acts as a platform to recruit these proteins. [240].

In addition, based on the results of above studies, it is complicated to establish if a particular device and direct or indirect exposure were instrumental in leading to direct DNA damage or to the triggering of cell death. In general, there are several difficulties in comparing the results of the variety of CAP studies using different parameters. The effect of CAP application also varies in different tumour types and cell lines. The voltage and power used in CAP application, the distance and on-and-off time of exposure are factors which should not be overlooked and their summarisation and comparison in the following studies can help determine the final CAP effects on cells—be it DNA damage or cell death.

Not only ROS and RNS, but also UV radiation can have detrimental effect on the DNA structure. It is recognised that UV radiation damages DNA, and especially UV-C (wavelength 100–280 nm) can be harmful [241,242]. However, the degree of damage depends also on dosage and exposure time, and CAP produces lower dosage of UV radiation compared to UV-light systems for sterilisation and low-pressure plasma systems [80,242,243,244]. Final dose of UV radiation generated by CAP also depends on various factors, such as carrier gas flow rate and generator intensity [80,242,245].

There has been reported direct effect of CAP-generated UV radiation on DNA damage and membrane disintegration due to etching in prokaryotes [246,247]. Heise et al. [248] and Trompeter et al. [249] presumed that DBDs efficiency of spore reduction using different gases—nitrogen, argon and synthetic air is related to the UV spectra of these gases in the discharge, and Heise et al. [248] reported under experimental conditions wavelengths below 280 nm when nitrogen was used as the working gas [248]. Park et al. [250], then reported that the intensity of UV light generated by the argon CAP ranged from 65 mW/cm^2^ to 94 mW/cm^2^ at 254 nm wavelength, and they also recorded this result in their following study [251]. Very recent study of Kletschus et al. [245] discussed the potential risk of UV-C emission during CAP application. The authors reported that the UV-C intensity can reach 124.5 ± 11 mW/m^−2^, depending on the distance maintained from the CAP flame, and a dose of 28.8 J/m^−2^ is incurred when irradiation lasts 240 s. This approximates the maximum 30 J/m^−2^ set for employees. In addition, the direct dosage for medical-dermatology procedures cannot exceed 3 mW/m^−2^ [245]. In this study, UV-C radiation emitted in all orientations in the area but was also shielded by the geometry of the handpiece. However, the conditions to cause this potential danger were specific, and the structure exposed to CAP had to be unmoved for at least the 4-min time limit.

Other researchers considered that there was negligible risk associated with CAP-generated UV-C radiation; (1) Perez et al. suggested that UV-C is efficiently absorbed in air, and that only UV-A reached the sample in their experimental regime [252]; (2) Choi et al. treated various bacteria with a DBD operated in air at atmospheric pressure, and they observed no UV radiation under 290 nm [253]. This was supported by (3) Laroussi et al. who recorded no UV under 285 nm with DBD device [254]; (4) Stoffels et al. [255] quantified UV emission between 250 and 400 nm, but the high intensities were reported only between 305 and 390 nm; (5) Plewa et al. [228] recorded no UV radiation below 290nm, and their highest intensities were from 337 to 357 nm, and although Gaur et al. [231] considered that CAP had a direct effect on DNA damage, their observed emissions were all in the UV-A spectrum.

Hermann et al. [256]. implemented a different CAP approach using the plasma jet in the treatment of *Bacillus globigii*. They blocked the reactive species flow with a quartz window, so that only UV radiation hit the target structures. These authors noted that their experiment made no difference to the number of viable bacteria. Finally, several studies demonstrated that CAP-generated UV radiation had little—or no—effect on oral mucosa defects under in vivo conditions [27,257,258].

Healthy eukaryotic cells have relatively effective protective and repair systems. These systems are relatively capable of removing pyrimidine dimers and other UV damage, provided the damage is not sustained [259]. Moreover, CAP application is rapid, lasting usually only seconds, or a few minutes. Additionally, many researchers considered that there is minimal risk of the shorter wavelength UV radiation from CAP causing DNA damage. However, in contrast, several authors suggest that UV radiation generated by CAP can have an ultimate effect on cells. In addition, the UV-A and UV-B can also harm cells in higher dosage [80], and final effect of CAP-generated UV is dependent on the specific plasma exposure parameters, such as the CAP devices employed and the experimental settings of each device [80,242,243,244]. Therefore, possible risks from UV radiation must be considered with every CAP application, and adequate adaptations implemented.

It has been reported that increased oxidative stress levels can cause abnormal DNA oxidation, with consequent chemical modification of the DNA bases [260,261]. Extensive and irreparable DNA oxidation can then lead to DNA breaks and cell death [260,261]. Guanine is most frequently oxidised with 8-oxo-7,8-dihydroguanine results (8-oxo-G), but the other three bases can also be affected. The 8-oxo-G can then bind to adenine instead of to cytosine [260,261], and the DNA double-strand helix can be distorted by disturbance of hydrogen bonding [262]. In addition, some authors suggest that DNA oxidation is an epigenetic modification which can modulate gene expression when it occurs in gene regulatory regions [263]. Okazaki et al. [264] and Attri et al. [262] demonstrated CAP effect on generation of 8-oxy-G in cell free conditions. These authors showed that both plasmids and cell free DNA had increased 8-oxo-G levels following CAP treatment. Okazaki et al. [264] added that CAP also caused an increase of 8-OxoG in ex vivo liver tissues. Attri et al. [262] then demonstrated that CAP treatment also oxidised amino acids to a great extent.

Further research on 8-oxo-G recorded; (1) human A549 cancer lung cells had increased 8-oxo-G levels after CAP exposure, and the cells had a greater number of consequent DNA breaks [265]; (2) Choi et al. demonstrated increased 8-oxo-G levels in the cytoplasm and nucleus of both A549 and SK-MEL 2 cells after CAP exposure [169]; (3) Kurita et al. [230] also recorded higher 8-oxo-G levels in the A549 lung cell lines and increased DNA strand breaks following CAP administration. Moreover, Kurita et al. [230] reported only slight loss of cancer cell viability after CAP treatment. They subsequently analysed the activity of the 8-oxo-G repair enzyme, and found it highly expressed after CAP irradiation.

Finally, Guo et al. expressed the interesting view-point [266] that CAP causes DNA damage in both prokaryotic cells and eukaryotic HeLA cell lines. They further considered that CAP should cause oxidative modification to proteins. Here, histones were considered the major ROS targets, and authors have reported that (1) these oxidative-modified histones then cross-linked to DNA and (2) other protein types, including scaffold proteins and chromatin and transcription regulators, could also be cross-linked to DNA. In support, this DNA/nuclear protein cross-linking has previously been demonstrated in cells with elevated levels of hydroxyl radicals [267].

## 9. Conclusions

CAP has great potential for wide use in modern medicine, especially in minimising patient trauma and burden. However, it is essential for analyses to better reveal the lesser-known aspects of CAP’s action at the molecular level, including its impact on epigenetic changes and on the expression and activity of non-coding RNA’s. Abnormalities in epigenetic changes and non-coding RNA activity and expression can have fatal impacts on cells, similar to those caused by altered cellular signalling and oncogene and tumour-suppressor gene expression.

CAP also has the following potential uses; (1) it can be used in synergistic treatment with other cancer pharmaceuticals, nanoparticles, enzymes, and other modern medicine methods including photodynamic therapy. CAP application can be especially beneficial in synergistic treatment with pharmaceuticals in reducing dosage amounts, but retaining efficacy in patient care; (2) CAP application can potentially replace irradiation which has deleterious side-affects and which must be applied precisely to avoid damaging nearby healthy cells, and (3) in vitro CAP application has been shown to influence signalling pathways which cause resistance to some drugs, and this research should now be expanded to in vivo conditions in animal models.

The development of devices able to apply CAP to internal structures will also prove beneficial, and further efforts should target improved techniques for the application of PAL. However, questions remain, especially those concerning CAP-produced free radicals and UV radiation effects on DNA damage. Although some studies identify the possibility that CAP can directly cause DNA damage, many more consider that the CAP-generated free radicals initially activate cascades proceeding towards cell death., and DNA damage and fragmentation then occur as the final death-knell. Evaluation of activity of other proteins included in DNA repair and the short-term kinetic measurements of γH2AX levels associated with apoptosis onset could help to better understand final effect of CAP on DNA damage. Finally, ongoing research should further assess CAP effects on lipid peroxidation, membrane etching and DNA oxidation These are important aspects associated with choosing the most appropriate type of CAP plasma device and its settings. The proposed advances in experimentation and knowledge will undoubtedly aid current understanding, and they should reveal the precise effects of CAP application on both healthy and tumour cells.

## Figures and Tables

**Table 2:** List of synergistically applied CAP with routine pharmaceuticals, chemotherapeutic agents, and nanoparticles.

Synergistic CAP Treatment with	Plasma Type/Gas Injected	Final Effect of This Combined Application	Exposure Time	Observed in Type of Cells/Tissue Analysed
Cisplatin [182]	Plasma jet/Argone	More significantly decreased tumour-cell viability than monotherapies, lower cis-platineconcentration required for Ic50	1–3 min	Oral squamous carcinoma cell lines SCC-15
Sulfasalazine [187]	Plasma jet/Helium	Higher apoptosis and DNA fragmentation rate; CAP effect is comparable to X-radiation; most significant reduction in intracellular GSH levels	2 min	Colorectal carcinoma HCT-116 cell lines
Tamoxifene [191]	Mesh-DBD	Restoration of sensitivity to tamoxifen by up to 50% in resistant cell lines; expression change in several genes connected with sensitivity restoration; assessment of *MX1* and *HOCX6* genes as mediators of this restoration	30 s every hour/10 times	Tamoxifen-resistant breast cancer MCF-7 cell lines, standard MCF7 cell lines
Doxorubicin, epirubicin, oxaliplatin and vorinostat [192]	kINPen ^®^ plasma jet/Argone	Achieved doxorubicin and epirubicin cytotoxic effect at significantly lower concentration, demonstration of role of cationic transporter SLC22A16 in increased cytotoxicity	30 s	Various melanoma cell lines—B16F0, B16F10, SK-MEL 28, MDA-MD231, MCF10A, PC-3, and SW480
Decarbazine [194]	Plasma jet/Argone	Most significant shrinkage of tumours in animal models, most significant increase in autophagy genes *LC3* and *ATG3* expression	45 s	Mouse melanoma B16 tumour cell lines, B16 tumour-bearing mice
Plasma-activated infusions with salinomycin [196]	Plasma jet/Helium	Stronger effect on tumour volume reduction and decreased metastasis potential compared to monotherapy, negative effect on mitochondrial network, induction of non-apoptotic cell death	1 and 5 min of PAI irradiation	Melanoma A2058 and 4 osteosarcoma cell lines, mouse osteosarcoma allografts LM8
Temozolomide [198]	Plasma jet/Helium	Enhancement of temozolomide cytotoxic effect in resistant cell lines with highly expressed *MGMT*	60 and 180 s	Glioblastoma U87MG cell lines
Photodynamic therapy [202]	Plasma jet/Helium	More significant cell viability decrease compared to monotherapies	60 s	Lung-cancer A549 cell lines
Gold nanoparticle/Gold nanoparticles bounded with FAK [206]	DBD device	Increase of the cell death-rate 2.5-fold (gold nanoparticles) and 5-fold (FAK bounded nanoparticles) compared to CAP monotherapy	40 s	Melanoma G361cell lines
PLGA nanoparticles [209]		Most significant viability reduction, CAP facilitated nanoparticle cell penetration and diminished microvilli and pseudopodia, down-regulation of *VEGF*, *MTDH*, *MMP2*, and *MMP9* genes	60 s	Breast cancer MDA-MB-231 cell lines
Gold nanoparticles [210]	DBD device fed with Helium	Increase in cell death by 30% compared to monotherapy	30 s	Glioblastoma U87 cell lines
Gold nanoparticles [212]	DBD device	Higher uptake of nanoparticles due to CAP action, significantly higher cytotoxic effect of combined therapy compared to monotherapy	30 s	Glioblastoma U373MG multiforme cells
Iron-oxide-based magnetic nanoparticles [214]	Plasma Jet/Helium	Most significant effect on cell viability reduction, inhibitory effect on cell migration, suppression of vimentin, stronger effect on VEGF pathway kinases phosphorylation, most notable reduction of tumour volume in animal models, inhibition of EMT	150 s	Lung-cancer A549 cell lines, mouse A549 xenografts
Lysozyme [217]	Pulsed DBD	Structural and functional change in lysozyme enzyme, lysozyme treated with CAP at pH 7 decreased tumour-cell viability more significantly, and this was associated with increased caspase activity	5,10,20 min	

CAP—Cold atmospheric plasma, DBD—Dielectric barrier discharge, EMT—Epithelial-to-mesenchymal transition, FAK—Focal Adhesion Kinase 1, GSH—Glutathione Synthase, Ic50—Half-maximal inhibitory concentration, min – minutes; PAI—Plasma-activated infusion, PLGA—Poly lactic-co-glycolic acid, s – seconds; VEGF—Vascular Endothelial Growth Factor.

## Data Availability

Not applicable.

## Abbreviations

ALA	5-Amino-levulinic acid
ASC	Adipose tissue-derived stem cells
BC	Breast cancer
BER	Base-excision repair
bp	Base pairs
CAP	Cold atmospheric plasma
DAC	Decarbazine
DBD	Dielectric barrier discharge
DBS	Double-strand breaks
DNMT	DNA methyl transferase
FE-DBD	Floating electrode dielectric barrier discharge
GSH	Intracellular-reduced glutathione
GNP	Gold nanoparticles
HAT	Histone acetylases
HDAC	Histone deacetylase
H2O2	Hydrogen peroxide
H3K4me	Histone 3 lysine 4 methylation,
H3K9me	Histone 3 lysine 9
H3K27me	Histone 3 lysine 27 methylation
H3K27me3	Histone 3 lysine 27 methylation trimethylation
°K	Kelvin
L•	Lipid radical
LOO•	Lipid peroxy-radical
lncRNA	Long non-coding RNA
MGMT	O6-methylguanine-DNA methyltransferase
MDA	Malondialdehyde
miR	MicroRNA
miRNA	MicroRNA
MNP	Iron-oxide-based magnetic nanoparticles
ncRNA	Non-coding RNA
NO	Nitric oxide
O_2_	Oxygen
PAL	Plasma-activated liquid
PAM	Plasma-activated medium
PARP1	Poly ADP-ribose polymerase-1
PTD	Photodynamic therapy
RNS	Reactive nitrogen species
ROS	Reactive oxygen species
SAL	Salinomycin
SSZ	Sulfasalazine
TET	Ten-eleven translocation methylcytosine dioxygenases
TMZ	Temozolomide
UV	Ultraviolet radiation
XR	X-radiation
γH2AX	Phosphorylated histone H2AX
4-HNE	4-hydroxy-2-nonenal
8-Oxo-G	8-oxo-7,8-dihydroguanine
